# Evaluation of Blood Glial Fibrillary Acidic Protein as a Potential Marker in Huntington's Disease

**DOI:** 10.3389/fneur.2021.779890

**Published:** 2021-11-19

**Authors:** Huajing You, Tengteng Wu, Gang Du, Yue Huang, Yixuan Zeng, Lishan Lin, Dingbang Chen, Chao Wu, Xunhua Li, Jean-marc Burgunder, Zhong Pei

**Affiliations:** ^1^Department of Neurology, The First Affiliated Hospital, Sun Yat-sen University, Guangdong Provincial Key Laboratory of Diagnosis and Treatment of Major Neurological Diseases, National Key Clinical Department and Key Discipline of Neurology, Guangzhou, China; ^2^Department of Neurology, Zhongshan Hospital, Fudan University, Shanghai, China; ^3^China National Clinical Research Centre for Neurological Diseases, Beijing Tiantan Hospital, Capital Medical University, Beijing, China; ^4^Centre for Neurological Diseases, Beijing Tiantan Hospital, Capital Medical University, Beijing, China; ^5^Department of Neurology, The First Affiliated Hospital of Shenzhen University, Health Center Shenzhen Second People's Hospital, Shenzhen, China; ^6^Swiss HD Centre, NeuroZentrumSiloah and Department of Neurology, University of Bern, Bern, Switzerland

**Keywords:** Huntington's disease, glial fibrillary acidic protein, neurofilament light protein, clinical severity, biomarker

## Abstract

**Objective:** Huntington's disease (HD) is an autosomal dominant neurodegenerative disorder. Neurofilament light protein (NfL) is correlated with clinical severity of HD but relative data are the lack in the Chinese population. Reactive astrocytes are related to HD pathology, which predicts their potential to be a biomarker in HD progression. Our aim was to discuss the role of blood glial fibrillary acidic protein (GFAP) to evaluate clinical severity in patients with HD.

**Methods:** Fifty-seven HD mutation carriers (15 premanifest HD, preHD, and 42 manifest HD) and 26 healthy controls were recruited. Demographic data and clinical severity assessed with the internationally Unified Huntington's Disease Rating Scale (UHDRS) were retrospectively analyzed. Plasma NfL and GFAP were quantified with an ultra-sensitive single-molecule (Simoa, Norcross, GA, USA) technology. We explored their consistency and their correlation with clinical severity.

**Results:** Compared with healthy controls, plasma NfL (*p* < 0.0001) and GFAP (*p* < 0.001) were increased in Chinese HD mutation carriers, and they were linearly correlated with each other (*r* = 0.612, *p* < 0.001). They were also significantly correlated with disease burden, Total Motor Score (TMS) and Total Functional Capacity (TFC). The scores of Stroop word reading, symbol digit modalities tests, and short version of the Problem Behaviors Assessments (PBAs) for HD were correlated with plasma NfL but not GFAP. Compared with healthy controls, plasma NfL has been increased since stage 1 but plasma GFAP began to increase statistically in stage 2.

**Conclusions:** Plasma GFAP was correlated with plasma NfL, disease burden, TMS, and TFC in HD mutation carriers. Plasma GFAP may have potential to be a sensitive biomarker for evaluating HD progression.

## Introduction

Huntington's disease (HD) is an autosomal dominant neurodegenerative disorder characterized by progressively deteriorative motor, psychiatric, and cognitive dysfunction. It is caused by abnormal expansion of CAG repeat in the huntingtin protein (HTT) gene (1). Although there are currently no treatments to cure HD, several disease-modifying therapies have shown some potential to slow HD progression (2, 3). However, objective measurements, such as biomarkers, are needed to precisely evaluate these novel disease-modifying interventions. In clinical practice, clinical rating scales, such as the internationally Unified Huntington's Disease Rating Scale (UHDRS), are widely used to assess disease severity, but those subjective scales may not be able to assess minor disease-related alterations in HD, especially in prodromal HD.

The most reliable biomarker for HD should be the mutant huntingtin protein (mHTT) in cerebrospinal fluid (CSF) but it has to be acquired by lumbar puncture (4). Recently, several blood-based biomarkers, such as plasma neurofilament light protein (NfL), have been evaluated in patients with HD. In European countries, NfL has been proposed as a promising biomarker to assess the therapeutic effect and track the progression in HD mutation carriers (5). NfL is the smallest subunit of neurofilaments and a major component of the neuronal cytoskeleton (6). Once axons of neurons in the brain are damaged, NfL is released into the CSF and blood. In HD mutation carriers, NfL is statistically correlated with clinical severity, CSF mHTT, and brain atrophy (7, 8). However, there is no information on plasma NfL in Chinese HD mutation carriers.

Although plasma NfL has shown a good ability to track HD progression, more biomarkers are required to better reflect HD progression comprehensively. Astrocytes are one of the most prevalent glial cell types in the mammalian brain (9). They are housekeepers of the brain and maintain brain function by regulating the maturation of synapses, neurotransmitter homeostasis, water and ion homeostasis, neurovascular coupling, and the formation of the blood-brain barrier (10). Astrocytes gradually lose their normal functions and become reactive in HD. Reactive astrocytes further boost neuroinflammation, which in turn drives neurodegeneration (10, 11). Moreover, astrocytes with nuclear mHTT inclusions also trigger oxidative stress in neurons (12). Indeed, reactive astrocytes are correlated with the severity of disease progression and striatal neurodegeneration in HD (13). Glial fibrillary acidic protein (GFAP) is an intermediate filament protein of astrocytes, and the expression of GFAP is increased in astroglial activation (9). Similar to NfL, GFAP can be released into CSF and blood. The potential role of blood GFAP as a biomarker has been explored in different brain diseases. For example, blood GFAP was reported to be associated with disease severity and MRI lesions in progressive multiple sclerosis (14). In addition, GFAP was also increased after mild traumatic brain injuries, suggesting the potential application of blood GFAP in neurological diseases (15). However, the role of blood GFAP in HD is lacking.

In this study, we aimed to evaluate the value of plasma NfL and GFAP related to clinical measurements in Chinese HD mutation carriers and explore the potential of plasma GFAP to be a biomarker for HD progression.

## Methods and Materials

### Participants and Study Design

A total of 57 HD mutation carriers (15 premanifest HD, preHD, and 42 manifest HD) from 44 families were recruited in the First Affiliated Hospital, Sun Yat-sen University from January 6, 2015 to November 18, 2019. Eight preHD participants from eight families were recruited in the Beijing Tiantan Hospital from May 8, 2019 to December 25, 2019. They were all definitely diagnosed by HTT CAG repeat expansion mutation (i.e., HTT CAG repeat expansion ≥ 40). Twenty-six healthy controls were recruited in this study, such as three family members without HD risk (i.e., spouses of HD mutation carriers) and nine controls with a family history of HD (i.e., three siblings and six offsprings of HD mutation carriers with a gene-expansion negative test). They were age matched to HD mutation carriers and clinically healthy. Individuals with vascular, infectious, inflammatory, or any other concomitant neurological disorders were excluded. Demographic data, clinical characteristics, and blood samples were collected from the participants. Correlations of clinical severity and plasma NfL/GFAP concentrations were assessed.

This study was compliant with the Declaration of Helsinki and approved by the ethics committees of the First Affiliated Hospital, Sun Yat-sen University, and Beijing Tiantan Hospital. The number of the approval is [2017]318. Written informed consent was obtained from each participant before enrollment.

### Clinical Assessment

Impairments of motor function and independent living skills were assessed with the UHDRS Total Motor Score (TMS) and UHDRS Total Functional Capacity (TFC), respectively. Cognitive function was evaluated with a series of tests, such as symbol digit modalities test, Stroop word reading test, Stroop color-naming test, Stroop interference test, trail making test, and category fluency test, as previously described (16). Neuropsychiatric characteristics were assessed using a short version of the Problem Behaviors Assessments (PBAs) for HD. Clinical stages were classified with UHDRS TMS and UHDRS TFC. If the score of UHDRS-TMS ≤ 5, the participant would be categorized as preHD. HD mutation carriers with the score of UHDRS-TMS > 5 were clarified as manifest HD, who would be further separated into four clinical stages (stage 1: 11 ≤ TFC ≤ 13; stage 2: 7 ≤ TFC ≤ 10; stage 3: 3 ≤ TFC ≤ 6; and stage 4: 1 ≤ TFC ≤ 2) (7). Disease burden was calculated as CAG age product (CAP) score: CAP = ([CAGn −33.66] × age) (17). Disease duration began from the initial onset of motor, psychiatric, or cognitive dysfunction. The clinical severity of all HD mutation carriers was investigated by two HD specialists, who were both UHDRS certified. The score of each item in UHDRS would be graded after discussion.

### Plasma NfL/GFAP Quantification

Two milliliter venous blood was collected from each participant with EDTA anticoagulation tubes (BD, Franklin Lakes, NJ, USA). Blood samples were centrifuged at 4,000 × g for 10 min at 4°C to remove hemocytes as soon as possible and stored in eppendorf tubes (Axygen, Union City, CA. USA) at −80°C. Samples were packed in dry ice for transportation and analysis. Plasma NfL and GFAP were quantified using an ultra-sensitive single-molecule (Simoa, Norcross, GA, USA) technology (Quanterix, Billerica, MA, USA) on the automated Simoa HD-1 platform (GBIO, Hangzhou, China) according to the instruction of manufacturer (18). The NF-light assay (Catalog number: 102258) and GFAP (Catalog number: 102336) kits were purchased from Quanterix and used accordingly. Plasma samples were diluted at a 1:4 ratio for both assays. Calibrators and quality controls were measured in duplicate. All sample measurements were performed on a single run basis. Limits of detection (LOD) and limits of quantification (LOQ) were also provided. Operators were unaware of the disease status of participants.

### Statistical Analysis

Original values are presented as mean ± SD. Concentrations of plasma NfL and GFAP were non-normally distributed because of biologically plausible higher values. Natural log-transformation produced an acceptable normal distribution, as previously reported (7). SPSS and GraphPad were used for statistical calculations (SPSS 19.0 software, Armonk, NY, USA; Prism 6, GraphPad, La Jolla, CA, USA). Spearman's rank correlation was performed between original plasma NfL/GFAP and log-transformed plasma NfL/GFAP. Potentially confounding demographic variables (age, gender, and CAG repeats) were examined in preliminary analyses and those found to be significant were included as covariates for subsequent analyses. ANOVA and multiple comparisons were used to compare plasma NfL/GFAP concentration between groups. Correlation between disease burden and plasma NfL/GFAP concentration was assessed with Pearson's correlation coefficient. Pearson's partial correlation using age, or age and CAG, as covariates was used to evaluate the linear correlation between plasma NfL and GFAP concentration or between analyte concentrations and clinical measures. The receiver operating characteristic (ROC) curve was used to analyze the diagnostic power of plasma NfL/GFAP for HD. Overall sensitivity and specificity were evaluated with areas under the curve (AUC). A cut-off value of each ROC curve was verified with the largest Youden index *p* < 0.05 was considered significant statistically.

## Results

### Demographic Features of all Participants

This study consists of 83 participants: 26 healthy controls, 15 preHD, and 42 manifest HD. They were all Chinese Han population. Demographic characteristics are presented in Table 1.

**Table 1:** Demographic features and intergroup comparison of plasma NfL/GFAP.

**Group**	**Control**	**PreHD**	**Manifest HD**
*n*	26	15	42
Age	35.73 ± 7.68	28.00 ± 7.53	44.19 ± 11.10
Gender (M/F)	15/11	8/7	24/18
CAG repeat expansion	N/A	43.13 ± 2.59	46.09 ± 4.44
Disease Burden	N/A	256.60 ± 60.99	519.10 ± 129.60
Plasma NfL (log pg/mL)	2.30 ± 0.50	2.69 ± 0.99	4.99 ± 0.64
Plasma GFAP (log pg/mL)	5.43 ± 0.54	5.77 ± 0.74	6.17 ± 0.74
Total functional capacity	N/A	13	9.12 ± 3.49
Total motor score	N/A	0.87 ± 1.60	52.79 ± 22.93
Disease duration (ys)	N/A	N/A	4.80 ± 4.41

*Mean values ± SD are shown for all demographic features of healthy control, preHD and manifest HD groups. NfL, neurofilament light protein; GFAP, glial fibrillary acidic protein; preHD, premanifest Huntington's disease; N/A, Not applicable*.

The mean age of healthy controls was 35.73 ± 7.68 years old, who were recruited to be age-matched to all HD mutation carriers. The preHD group was significantly younger than the manifest HD group (44.19 ± 11.10 vs. 28.00 ± 7.53, *p* < 0.0001). There were no intergroup differences in gender. The mean CAG repeat expansion was 45.32 ± 4.27 in HD mutation carriers, ranging from 40 to 58. Disease burden was lower in the preHD group than in the manifest HD group (256.60 ± 60.99 vs. 519.10 ± 129.60, *p* < 0.0001). There were 20, 11, 10, and 1 manifest HD participants in stages 1, 2, 3, and 4, respectively. The full score of UHDRS-TFC was obtained in all preHD and stage 1 of some manifest HD participants. The disease duration of the manifest HD group was 4.80 ± 4.41 years, ranging from 1 to 20 years. Plasma NfL and GFAP concentrations were quantified in all participants and analyte concentrations by group are shown in Table 1. The correlation between original plasma NfL/GFAP and log-transformed NfL/GFAP was performed (Spearman's rank correlation coefficient: NfL, 1.000, *p* < 0.0001; GFAP, 1.000, *p* < 0.0001).

### Plasma GFAP Was Correlated With Plasma NfL in HD

**Figure 1:**
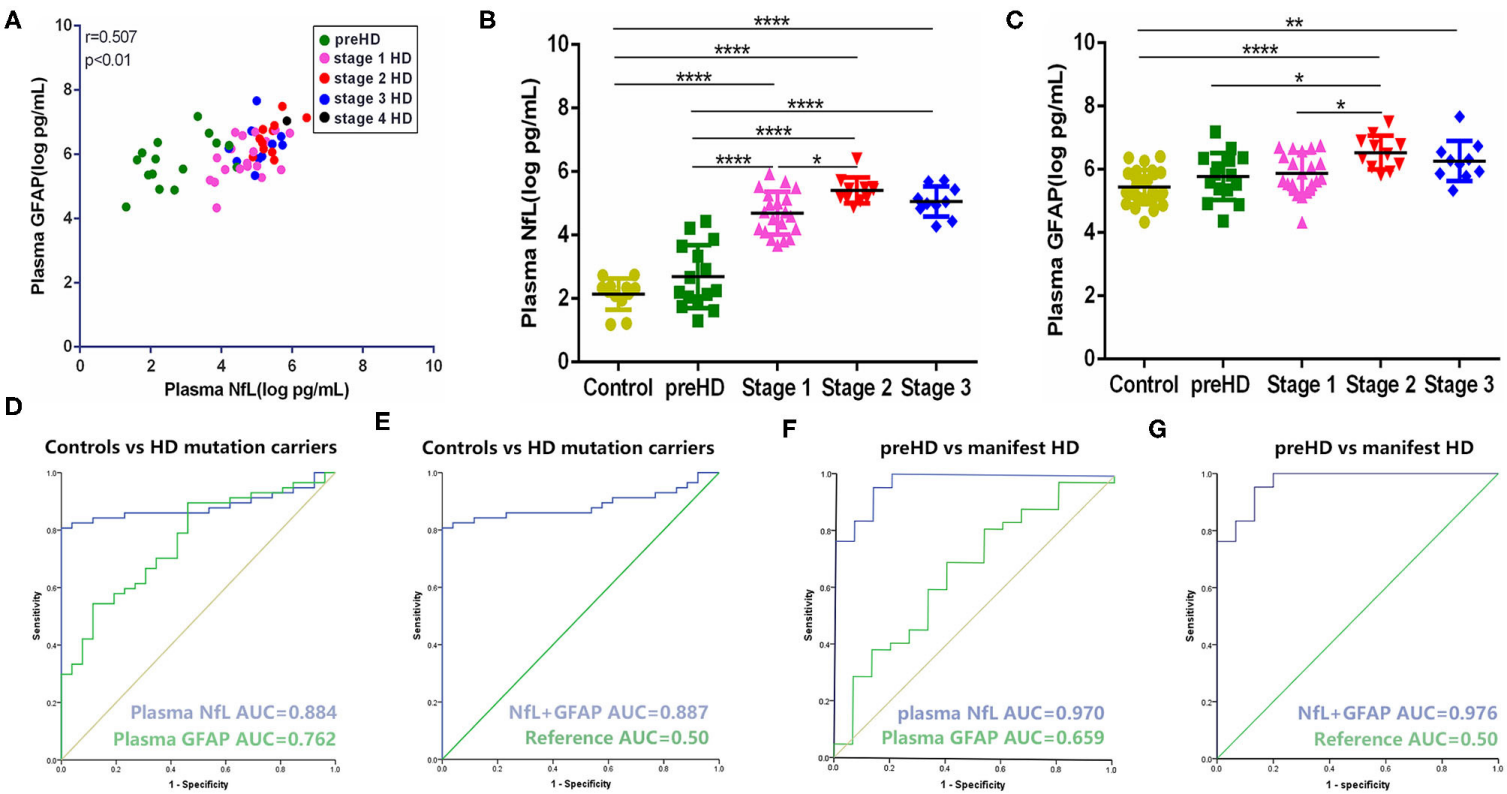
Comparison and correlation of plasma NfL and GFAP. **(A)** Correlation of plasma NfL and GFAP in HD mutation carriers (*n* = 57). **(B)** Concentration of plasma NfL across disease stages (controls: *n* = 26; preHD: *n* = 15; stage 1: *n* = 20; stage 2: *n* = 11; stage 3: *n* = 10). **(C)** Concentration of plasma GFAP across disease stages (controls: *n* = 26; preHD: *n* = 15; stage 1: *n* = 20; stage 2: *n* = 11 stage 3: *n* = 10). **(D)** ROC curves for discrimination between controls (*n* = 26) and HD mutation carriers (*n* = 57) [95% confidence intervals (CIs) for AUCs: plasma NfL, 0.810–0.958, *p* < 0.0001; plasma GFAP, 0.657–0.868, *p* < 0.0001]. **(E)** ROC curves for discrimination between controls (*n* = 26) and HD mutation carriers (*n* = 57) with combination of plasma NfL and GFAP (95% CIs for AUCs: 0.814–0.960, *p* < 0.0001). **(F)** ROC curves for discrimination between preHD (*n* = 15) and manifest HD participants (*n* = 42) (95% CIs for AUCs: plasma NfL, 0.929–1.000, *p* < 0.0001; plasma GFAP, 0.496–0.822, *p* = 0.070). **(G)** ROC curves for discrimination between preHD (*n* = 15) and manifest HD participants (*n* = 42) with combination of plasma NfL and GFAP (95% CIs for AUCs: 0.934–1.000, *p* < 0.0001). Significance level was defined as *p* < 0.05. ^*^p < 0.05, ^**^p < 0.01, and ^****^p < 0.0001. NfL, neurofilament light protein; GFAP, glial fibrillary acidic protein; HD, Huntington's disease; ROC, receiver operating characteristic.

In our study, the trends of plasma NfL and GFAP were similar along with the disease progression. They have a strong linear positive correlation with each other (*r* = 0.612, *p* < 0.001; Figure 1A). Compared with healthy controls, plasma NfL and GFAP began to increase significantly in stages 1 and 2, respectively. For preHD participants, concentrations of both plasma NfL and GFAP did not differ significantly from those of healthy controls (healthy controls vs. preHD, NfL: 2.30 ± 0.50 vs. 2.69 ± 0.99 log pg/ml, *p* > 0.05; GFAP: 5.43 ± 0.54 vs. 5.77 ± 0.74 log pg/ml, *p* > 0.05; Figures 1B,C). For manifest HD participants, NfL but not GFAP was significantly increased in stage 1 compared with premanifest ones (preHD vs. stage 1, NfL: 2.69 ± 0.99 vs. 4.69 ± 0.68 log pg/ml, *p* < 0.0001; GFAP: 5.77 ± 0.74 vs. 5.88 ± 0.65 log pg/ml, *p* > 0.05). However, the differences between stages 1 and 2 in plasma NfL and GFAP were both significant (stage 1 vs. stage 2, NfL: 4.69 ± 0.68 vs. 5.41 ± 0.41 log pg/ml, *p* < 0.05; GFAP: 5.88 ± 0.65 vs. 6.53 ± 0.53 log pg/ml, *p* < 0.05). Plasma NfL and GFAP were slightly lower in stage 3 but the differences were not significant (stage 2 vs. stage 3, NfL: 5.41 ± 0.41 vs. 5.06 ± 0.48 log pg/ml, *p* > 0.05; GFAP: 6.53 ± 0.53 vs. 6.26 ± 0.54 log pg/ml, *p* > 0.05). There was only one HD participant in stage 4. The concentrations of plasma NfL and GFAP in stage 4 HD were 5.85 and 7.04 log pg/ml, respectively. LOD and LOQ were much lower than the concentrations of our samples.

Compared with plasma NfL, plasma GFAP exhibited less sensitivity (cut-off value: 0.433 vs. 0.807) and smaller AUC (0.762 vs. 0.884) in terms of distinguishing healthy controls and HD mutation carriers (Figure 1D). Given the strong correlation of GFAP with NfL in HD, we further tested whether the combination of NfL and GFAP can synergistically increase the diagnostic power of HD. Unexpectedly, the combination of NfL and GFAP did not significantly increase the diagnostic power (Figure 1E). We also tested the ROC curve in terms of distinguishing premanifest and manifest HD participants. Plasma NfL showed remarkable diagnostic ability [AUC = 0.970, 95% CIs for AUC: 0.929–1.000, *p* < 0.0001] while plasma GFAP could not distinguish preHD and manifest HD (AUC = 0.659, 95% CIs for AUC: 0.496–0.822, *p* = 0.070; Figure 1F). Similarly, the combination of NfL and GFAP did not significantly increase the diagnostic power of distinguishing premanifest and manifest HD participants (AUC = 0.976, 95% CIs for AUC: 0.934–1.000, *p* < 0.0001; Figure 1G).

### Plasma NfL Had a Strong Correlation With Clinical Severity

**Figure 2:**
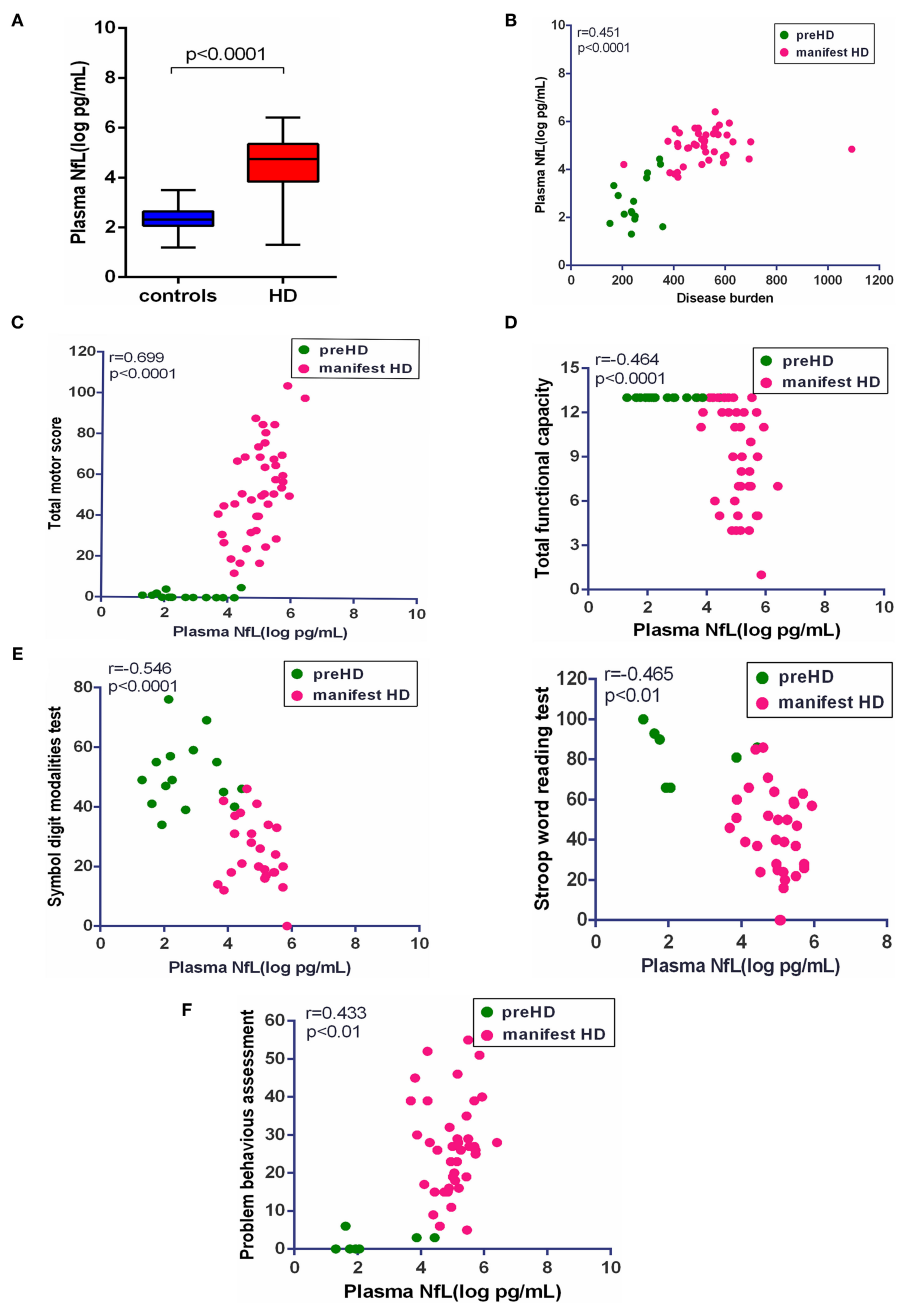
Correlation of plasma NfL and clinical measures. **(A)** The concentration of plasma NfL in healthy controls (*n* = 26) and HD mutation carriers (*n* = 57). **(B)** Plasma NfL was significantly correlated with disease burden in HD mutation carriers. **(C)** Plasma NfL was significantly correlated with Total Motor Score in HD mutation carriers. **(D)** Plasma NfL was significantly correlated with Total Functional Capacity in HD mutation carriers. **(E)** Plasma NfL was significantly correlated with the scores of symbol digit modalities and Stroop word reading tests in HD mutation carriers. **(F)** Plasma NfL was significantly correlated with the score of the short version of the Problem Behaviors Assessment for HD. Scatter plots show unadjusted values. R and *p* values are age-adjusted, generated from Pearson's partial correlations including age as a covariate. The significance level was defined as *p* < 0.05. NfL, neurofilament light protein.

In our study, plasma NfL was significantly higher in HD mutation carriers than healthy controls (healthy controls vs. HD mutation carriers, 2.30 ± 0.50 vs. 4.39 ± 1.26 pg/ml, *p* < 0.0001; Figure 2A). In terms of HD mutation carriers, plasma NfL was statistically correlated with disease burden (*r* = 0.451, *p* < 0.0001, Figure 2B).

The clinical assessments were conducted in 57 HD mutation carriers. All HD mutation carriers completed UHDRS TMS and TFC. Some participants failed to complete PBAs or cognitive assessments (Supplementary Table 1). Among these clinical measures, the scores of TMS (*r* = 0.699, *p* < 0.0001), TFC (*r* = −0.464, *p* < 0.01), symbol digit modalities (*r* = −0.546, *p* < 0.0001), Stroop word reading tests (*r* = −0.465, *p* < 0.01), and PBAs (*r* = 0.433, *p* < 0.01) were all significantly correlated with the concentration of plasma NfL (Figures 2C–F). However, the residual clinical measures had no correlation with plasma NfL. All the results were age-adjusted. If using age and CAG as a covariate, concentration of plasma NfL was also correlated with the scores of TMS (*r* = 0.400, *p* < 0.01), symbol digit modalities (*r* = −0.380, *p* < 0.05), and PBAs (*r* = 0.384, *p* < 0.01; Table 2).

**Table 2:** Association between plasma NfL/GFAP and clinical measures in HD mutation carriers.

**Clinical measures**	**Adjusted for**	**Plasma NfL**		**Plasma GFAP**	
		***r***	***P* value**	***r***	***P* value**
Total motor score	Age	0.699	**<0.** **0001**	0.286	**<0.05**
	Age and CAG	0.400	**<0.01**	0.175	>0.05
Total functional capacity	Age	−0.464	**<0.0001**	−0.323	**<0.05**
	Age and CAG	−0.112	>0.05	−0.238	>0.05
Stroop word reading test	Age	−0.465	**<0.01**	−0.052	>0.05
	Age and CAG	−0.380	**<0.05**	−0.019	>0.05
Symbol digit modalities test	Age	−0.546	**<0.0001**	−0.107	>0.05
	Age and CAG	−0.159	>0.05	0.157	>0.05
Short version of the problem behavior assessment for HD	Age	0.433	**<0.01**	0.216	>0.05
	Age and CAG	0.384	**<0.01**	0.157	>0.05

*R and p values are generated by Pearson's partial correlation including age, or age and CAG, as covariates. Significant associations are highlighted in bold and underline. NfL, neurofilament light protein; GFAP, glial fibrillary acidic protein*.

### Plasma GFAP Was Correlated With Clinical Severity

**Figure 3:**
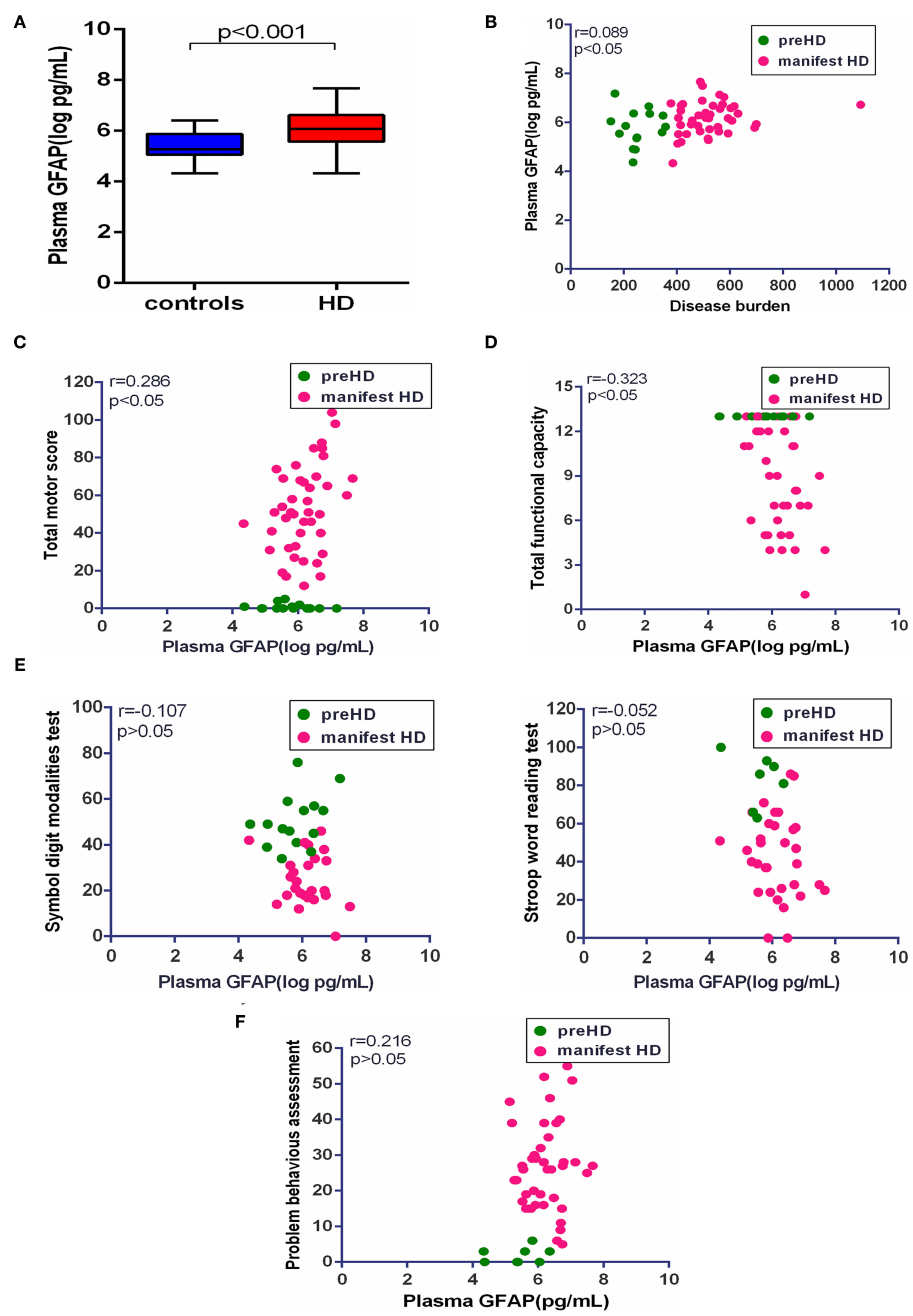
Correlation of plasma GFAP and clinical measures. **(A)** The concentration of plasma GFAP in healthy controls (*n* = 26) and HD mutation carriers (*n* = 57). **(B)** Plasma GFAP was significantly correlated with disease burden in HD mutation carriers. **(C)** Plasma GFAP was significantly correlated with Total Motor Score in HD mutation carriers. **(D)** Plasma GFAP was significantly correlated with Total Functional Capacity in HD mutation carriers. **(E)** Plasma GFAP had no correlation with the scores of symbol digit modalities and Stroop word reading tests in HD mutation carriers. **(F)** Plasma GFAP had no correlation with the score of the short version of the Problem Behaviors Assessment for HD. Scatter plots show unadjusted values. R and *p* values are age-adjusted, generated from Pearson's partial correlations including age as a covariate. The significance level was defined as *p* < 0.05. GFAP, glial fibrillary acidic protein.

Consistent with plasma NfL, plasma GFAP was significantly higher in HD mutation carriers compared with healthy controls (healthy controls vs. HD mutation carriers, 5.43 ± 0.54 vs. 6.07 ± 0.70 pg/ml, *p* < 0.01; Figure 3A). The correlation between plasma GFAP and disease burden was also linearly positive (*r* = 0.089, *p* < 0.05, Figure 3B). Among the above clinical measures, TMS (*r* = 0.286, *p* < 0.05) and TFC (*r* = −0.323, *p* < 0.05) were both significantly correlated with plasma GFAP (Figures 3C,D). However, the concentration of plasma GFAP had no correlation with the scores of all cognitive assessments and PBAs (Figures 3E,F). All the results were age adjusted. Plasma GFAP showed no correlation with the above clinical measures using age and CAG as a covariate (Table 2).

## Discussion

Huntington's disease progression should be evaluated precisely by CSF mHTT but it is hard for patients with HD to cooperate. The signal obtained by the CSF mHTT assay was also influenced by the somatic instability of CAG repeat length (5). In this retrospective cohort study, we found that NfL was significantly correlated with clinical severity, which was in accordance with previous literatures based on the European population (5, 7). Therefore, our data further support NfL as a promising biomarker to predict disease onset, progression, and treatment response in Chinese HD mutation carriers. In addition, we found that plasma GFAP was significantly increased in HD mutation carriers. Plasma GFAP and NfL were significantly correlated and showed similar trends overall. GFAP is a hallmark protein of astrocytes and has been widely used as the standard marker of astrocytic reactivity (19). In patients with HD, reactive astrocytes are strongly related to disease progression (20). Consistently, plasma GFAP was significantly correlated with disease severity and clinical stages, indicating the potential of GFAP as a biomarker of disease progression.

Astrocytes are implicated in cell loss or dysfunction of striatal neurons in HD (13). As the most abundant cell type in the brain, astrocytes not only provide support to neurons but also have an active role in brain functions (21). Thus, astrocytes may be a potential drug target in HD. Indeed, treatments targeting astrocytes have shown to be beneficial. For example, targeting neurotoxic reactive A1 astrocytes may revise the neurodegeneration and repair the function of neurons in HD (22). Additionally, we found that reactive astrocytes impeded the delivery of antisense oligonucleotides (ASOs) to deeper brain structures whereas inhibition of reactive astrocytes could increase the efficacy of ASOs in transgenic HD animals (23). In clinical practice, disease burden calculated by CAP score, an index of cumulative toxicity of mHTT, is used to estimate the proximity to HD diagnosis and reflect the severity of striatal dysfunction (24, 25). In the present study, GFAP was well correlated with NfL in terms of disease burden and clinical severity. Both NfL and GFAP showed significant diagnostic efficacy when distinguishing healthy controls and HD mutation carriers. However, plasma NfL but not GFAP showed significant diagnostic efficacy in terms of distinguishing preHD and manifest HD participants. In addition, the combination of NfL and GFAP did not significantly increase the diagnostic accuracy in both cases either. This indicated that plasma NfL maybe a more sensitive biomarker to reflect the accumulation of mHTT toxicity in HD pathological processes.

The symptoms of HD are largely driven by selective striatal neuronal loss in the early stages of HD. Pathologically, GFAP positive astrocytes were absent even when the striatal neuronal loss had begun in the HD human brain (13, 26, 27). Consistently, GFAP concentration was not significantly increased in the early stages of HD. We found that both plasma NfL and GFAP were significantly correlated with TMS, a score for motor impairment. It is the first time to study the association of PBAs with plasma biomarkers in HD. Neuropsychiatric changes are very common in HD and maybe present many years before motor clinical symptoms. PBAs were developed to measure the severity and frequency of behavioral symptoms in HD. However, there is limited information on neurobiological evidence for PBAs in HD. In the present study, PBAs were correlated with NfL, indicating a direct association of PBAs with neuronal damage. Neuropsychiatric symptoms were believed to arise from striatal pathology, which led to a disruption in striato-cortical circuits (28). Progressive neurodegeneration of specific cortical-subcortical circuitry would give rise to the deterioration of neuropsychiatric symptoms (29). Nevertheless, the association of PBAs with NfL needs further validation in large cohort studies.

In the present study, GFAP varied widely in preHD participants. GFAP levels in some preHD participants were as high as in middle stage. Recently, several fluid biomarkers have been developed to assess reactive astrocytes in the central nervous system including S100B (a calcium-binding protein) and YKL-40 (Chitinase 3-like 1) (30, 31). For example, elevated YKL-40 has been reported in preHD patients. A combination of these biomarkers may help understand the role of astrocytes in HD pathology and create a better prediction model.

There are some limitations in our study. First of all, preHD and control groups of our study were relatively small and this limited our ability to determine the earliest alterations in both NfL and GFAP. We also need more manifest HD participants to verify the clinical significance of plasma GFAP. Secondly, longitudinal follow-up research should be performed to track the dynamic change of plasma GFAP with disease course and explore its ability to predict disease progression. Thirdly, no juvenile HD participants were included in our study, which helps to understand whether they display similar or different profiles for NfL and GFAP.

## Conclusions

In conclusion, we identified a significantly elevated concentration of plasma NfL and GFAP in Chinese HD mutation carriers. It is the first time to explore the potential of plasma GFAP in HD progression. This study provided evidence for the promising application of plasma GFAP in predicting clinical severity.

## Data Availability Statement

The raw data supporting the conclusions of this article will be made available by the authors, without undue reservation.

## Ethics Statement

The studies involving human participants were reviewed and approved by the Ethics Committees of the First Affiliated Hospital, Sun Yat-sen University and Beijing Tiantan Hospital. The patients/participants provided their written informed consent to participate in this study.

## Author Contributions

HY, TW, J-mB, and ZP conceived the conception and designed the research. HY, TW, GD, YZ, and LL performed the acquisition, analysis, and interpretation of data. DC, CW, XL, and YH acquired data of HD mutation carriers. HY and ZP drafted the manuscript. ZP revised the manuscript and approved the final version of the manuscript. All authors contributed to the article and approved the submitted version.

## Funding

This work was supported by the National Natural Science Foundation of China (Grant Numbers 81873751 and 82071255), the National Key Research and Development Program of China (Grant Number 2017YFA0105104), the Guangdong Provincial Key Laboratory of Diagnosis and Treatment of Major Neurological Diseases (2020B1212060017), Guangdong Provincial Clinical Research Center for Neurological Diseases (2020B1111170002), the Southern China International Cooperation Base for Early Intervention and Functional Rehabilitation of Neurological Diseases (2015B050501003 and 2020A0505020004), Guangdong Provincial Engineering Center For Major Neurological Disease Treatment, Guangdong Provincial Translational Medicine Innovation Platform for Diagnosis and Treatment of Major Neurological Disease.

## Conflict of Interest

The authors declare that the research was conducted in the absence of any commercial or financial relationships that could be construed as a potential conflict of interest.

## Publisher's Note

All claims expressed in this article are solely those of the authors and do not necessarily represent those of their affiliated organizations, or those of the publisher, the editors and the reviewers. Any product that may be evaluated in this article, or claim that may be made by its manufacturer, is not guaranteed or endorsed by the publisher.
