# Supplementary material for: Evaluation of Blood Glial Fibrillary Acidic Protein as a Potential Marker in Huntington's Disease
Source: Front Neurol. 2021 Nov 19;12:779890. doi: 10.3389/fneur.2021.779890 (PMC8639701; doi:10.3389/fneur.2021.779890)
Supplement: Supplementary file 1 [file Table_1.DOCX]

**Supplementary Table 1** Number of participants in clinical measures

| Clinical measures | preHD | Manifest HD | Total account |
| --- | --- | --- | --- |
| Total motor score | 15 | 42 | 57 |
| Total functional capacity | 15 | 42 | 57 |
| Stroop word reading test | 7 ^#^ | 32 ^*^ | 39 |
| Symbol digit modalities test | 15 | 25 ^*^ | 39 |
| Short version of the Problem Behavious Assessment for HD | 7 ^#^ | 42 | 49 |

^#^ 8 preHD participants from the Beijing Tiantan Hospital didn’t performed stroop word reading test and short version of the Problem Behavious Assessment for HD.

* 10 and 17 manifest HD participants in stage 2-3 failed to understand the rules of stroop word reading test and symbol digit modalities test so they didn’t performed these two tests, respectively.
